# Activated Carbon-Enriched Electrospun-Produced Scaffolds for Drug Delivery/Release in Biological Systems

**DOI:** 10.3390/ijms24076713

**Published:** 2023-04-04

**Authors:** Zhanna K. Nazarkina, Alena O. Stepanova, Boris P. Chelobanov, Ren I. Kvon, Pavel A. Simonov, Andrey A. Karpenko, Pavel P. Laktionov

**Affiliations:** 1 Institute of Chemical Biology and Fundamental Medicine, Siberian Branch, Russian Academy of Sciences, 630090 Novosibirsk, Russia; zha_naz@niboch.nsc.ru (Z.K.N.); alena.o.lebedeva@gmail.com (A.O.S.); boris.p.chelobanov@gmail.com (B.P.C.); 2 Meshalkin National Medical Research Center, Ministry of Health of the Russian Federation, 630055 Novosibirsk, Russia; andreikarpenko@rambler.ru; 3 Department of Natural Sciences, Novosibirsk State University, 630090 Novosibirsk, Russia; spa_nsk@mail.ru; 4 Boreskov Institute of Catalysis, Siberian Branch, Russian Academy of Sciences, 630090 Novosibirsk, Russia; kvon@catalysis.ru

**Keywords:** activated carbon, controlled release, drug delivery, electrospinning, sirolimus

## Abstract

To vectorize drug delivery from electrospun-produced scaffolds, we introduce a thin outer drug retention layer produced by electrospinning from activated carbon nanoparticles (ACNs)-enriched polycaprolacton (PCL) suspension. Homogeneous or coaxial fibers filled with ACNs were produced by electrospinning from different PCL-based suspensions. Stable ACN suspensions were selected by sorting through solvents, stabilizers and auxiliary components. The ACN-enriched scaffolds produced were characterized for fiber diameter, porosity, pore size and mechanical properties. The scaffold structure was analyzed by scanning electron microscopy and X-ray photoelectron spectroscopy. It was found that ACNs were mainly coated with a polymer layer for both homogeneous and coaxial fibers. Drug binding and release from the scaffolds were tested using tritium-labeled sirolimus. We showed that the kinetics of sirolimus binding/release by ACN-enriched scaffolds was determined by the fiber composition and differed from that obtained with a free ACN. ACN-enriched scaffolds with coaxial and homogeneous fibers had a biocompatibility close to scaffold-free AC, as was shown by the cultivation of human gingival fibroblasts and umbilical vein cells on scaffolds. The data obtained demonstrated that ACN-enriched scaffolds had good physico-chemical properties and biocompatibility and, thus, could be used as a retaining layer for vectored drug delivery.

## 1. Introduction

Targeted drug delivery is a common problem of medical therapy. It becomes more acute when delivering drugs to specialized tissues and cells, for example, cytotoxic agents in tumor cells [1]. The development of tissue engineering and the production of constructs for tissues [2], or even organ [1] replacement, demand topographically specific local drug delivery. Local drug delivery could help to prevent scar tissue growth by various types of meshes, bacterial growth on tissue-engineered constructs and unwanted cell proliferation and inflammation. The tissue engineering structures should be seeded with different types of cells (before or after implantation) and have contact with different tissues and cells during implantation [3]. To provide conditions for the proliferation of a selected cell type in such constructs, targeted and local drug delivery (using low-weight compounds, such as valproic acid [4], or higher molecules, such as DNA, mRNA or microRNA [5]) is demanded. Local drug delivery is also concerned with drug saving and the prevention of unwanted systemic drug circulation, which causes side effects. It should be noted that poorly targeted drug delivery necessitates drug overdosing, which can induce subsequent undesired effects concerning non-drug-saving protocols. For example, paclitaxel-coated balloons showed a significant increase in long-term mortality compared to balloon angioplasty and bypass [6], which was apparently due to the high drug concentration released into the bloodstream.

Drug overdose can also be reduced by properly driving drug delivery kinetics, i.e., optimizing the release of a deposited drug from tissue-engineered constructs, which is an important aspect of drug delivery. It should be mentioned that all drugs have a threshold dose, an ED50 dose and a maximum dose. If one goes over the maximum dose, the effect plateaus or the drugs become toxic, so exceeding the maximum dose is pointless. From another point of view, higher doses must have a longer effect and are welcome. The local drug concentration at the implantation site decreases due to the drug’s distribution, circulation and excretion. A longer effect could be achieved with the gradual release of the drug in sufficient concentration from the deposits. At the same time, it is necessary to take into account the rate of diffusion of the drug in the tissues to support a threshold dose. On the other hand, drug-enriched materials release drugs by a diffusion-dependent [7] or diffusion-independent [8] mechanism. In both cases, the amount of drug released over time depends on the initial concentration of the drugs deposited in the material. Considering the aforementioned ideas on the effective drug concentration, one can claim that a tissue-engineered construct must contain drugs in amounts sufficient for the long-term functioning of the construct (or the time of its engraftment), but released in reasonable doses for a long time. These demands are in conflict with each other, and thus, novel approaches for managing drug release are demanded. The use of drug-loaded activated carbon (AC) reduces the concentration and the side effects of the drug. In vitro and murine model studies revealed that acyclovir-encapsulated carbon reduced the dosing frequency and demonstrated greater therapeutic efficacy than approved topical or systemic acyclovir alone [9].

Filling fibers with AC appears to be a promising approach for targeted or long-term drug delivery from electrospun fibers. AC-enriched scaffolds, produced by electrospinning (ES), were offered for drinking water production [10]; the adsorption of metal ions, such as Cr(VI) [11], As(III) [12] and Cu(II) [13]; the elimination of dyes, such as methyl orange [11], methylene blue and Congo red [14]; removing natural organic molecules from different solutions [15]; and for oil spill treatment [16]. AC-enriched scaffolds were tested as filters [17] from chemical and biological warfare agents and as protective clothing [18]. Enrichment of the scaffolds with AC was also shown to increase UV-shielding properties [19]. Many studies apply AC in polyacrylonitrile or cellulose acetate nanofibers and intended for the extraction of pollutants from environmental samples [10,11,12,13,14,15,16]. When packed in the previously mentioned polymers, as well as polystyrene and poly(L-lactide), AC retains its adsorption capacity [10,19]. In some studies, scaffolds and adsorption were conscientiously studied [11].

However, AC-enriched scaffolds were not studied in biological fluids as drug carriers for prolonged drug delivery or as an approach to vectorized drug delivery. Yet, there are a lot of AC types with different surfaces and pores that are able to bind an infinite set of organic molecules or biomolecules. Previously, we found that grinding AC up to 100 nm particles did not drastically change its binding capacity and could be used for filling ES-produced fibers [20]. One of the applications of drug-eluting scaffolds is the coating of vascular stents. We previously demonstrated that vascular stents coated by electrospinning with drug-eluting scaffolds were superior to bare-metal stents [21]. The dose of the drug in such coatings should be sufficient for reliable long-term inhibition of neointimal growth, and at the same time, the side effects of the drug should be minimal. The side effects are mainly associated with the drug entering the bloodstream. At that point, increasing the drug release period, supporting an efficient drug concentration in the vascular wall, and the prevention of drug release in the systemic circulation are demanded.

To vectorize drug release in the vascular wall and prevent its pulse influx in the systemic circulation, we propose incorporating a drug retention layer enriched with activated carbon nanoparticles into electrospun scaffolds. Drug release from ACN-enriched fiber scaffolds loaded with drugs in turn can be used to prolong drug delivery. Here, the efficacy of drug absorption/release by ACN-enriched scaffolds in conditions similar to those in living organisms was studied.

## 2. Results and Discussion

### 2.1. Preparation of ACN Suspensions

**Figure 1:**
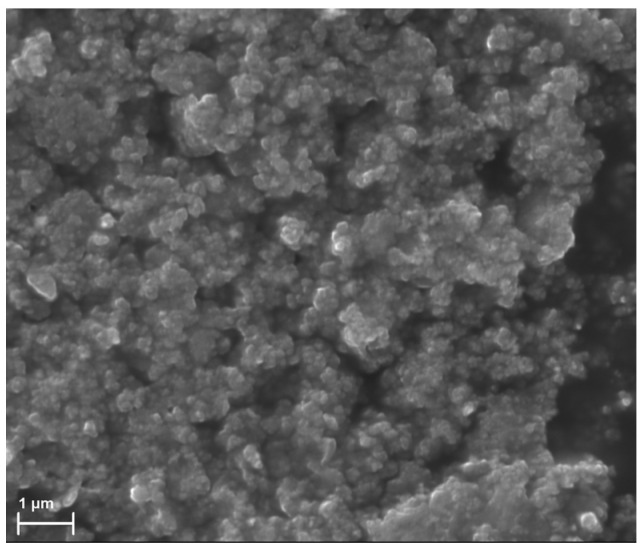
SEM image of ACN fraction after grinding with a bead mill.

For the purpose of the present work, activated carbon AX-21 with a surface area, determined by the Brunauer–Emmett–Teller method (BET), and pore volume of 2330 m^2^/g and 1.7 cm^3^/g, respectively [22], was ground as described in [19] to obtain 100 nm-sized AC particles. The physicochemical properties of ground ACNs are described in detail in [20]. Prior research has shown that the fiber diameter and orientation affect cell adhesion and proliferation [23,24,25]. In order to pack AC into electrospun fibers ~1 µm in diameter, the carbon particle sizes must be less than 0.3 μm. According to the data presented in Figure 1, the AC fraction after grinding with a bead mill is composed of particles in a range of 100 nm that can be packed into fibers of higher diameter.

ACN suspensions were prepared with conditions leading to the disintegration of the particles and the reduction of air bubbles in the pores, as described in 3.4. Electrospinning suspensions must be stable for at least a time interval required to produce a scaffold. The ability of solvents (ethanol, 1,1,1,3,3,3-hexafluoroisopropanol (HFIP), trifluoroethanol (TFE) and water) and suspension stabilizers (polyvinylpyrrolidone (PVP), polyethylene glycol (PEG), polyvinyl alcohol (PVA) and water) to disperse ACNs and support suspensions was assessed by the appearance of the solution and the tendency for rapid (30 min) settling of the suspension. The stability of the ACN suspensions was investigated over long time intervals, and the settling of ACNs in less than 24 h was classified as semistable, while settling in more than 24 h was classified as stable. The properties of the suspensions are summarized in Table 1. It should be noted that the density of the solvent used for particle flotation is of great importance, as the use of ethanol and water (densities of 0.789 and 1, respectively) is clearly less convenient compared to TFE and HFIP (densities of 1.393 and 1.605, respectively). According to Table 1, ethanol and water are the worst solvents for the preparation of stable ACN suspensions. The most stable suspensions contained 2.5% ACNs in a 5–10% PCL solution in HFIP and 5% ACNs in a 5% PVP solution in HFIP. PVP is a non-toxic and biocompatible chemical that is used in pharmaceutical formulations and as a blood substitute solution compound [26], making it suitable for use in implantable medical devices. These data were taken into account when suspensions were produced for the fabrication of ACN-enriched scaffolds.

The suspensions of ACNs are, nevertheless, prone to forming aggregates that are difficult to disintegrate. Even if the suspension looks stable, it has a tendency for slow precipitation and the formation of a down layer with higher ACN concentrations. The preparation of fibers containing ACNs completely coated with polymer demands a suspension free of large aggregates.

To find out the conditions for the removal of large-size particles from the suspensions, we used subsequent centrifugation steps with gravimetric measurements of the pellets (Table 2). The fraction of smaller particles, which forms stable ACN suspensions, was reached by centrifugation of the suspension at 1000× *g* for 1 min. The ACN losses after centrifugation were comparable for suspensions in PVP and PCL (11.5% and 10.8%, respectively). AC particles ground with the bead mill in the PVP solution tended to aggregate to a lesser extent than the AC particles ground in water. The centrifugation of suspensions for 0.5 and 1 min and subsequent centrifugation of 0.5 + 1 min resulted in the sedimentation of large aggregates, according to microscopy. Yet, centrifugation for only 0.5 min led to unequal feeding of the suspension through the needle. Despite the ACN losses, non-settling/non-separating ACN suspensions were obtained for all subsequent experiments by centrifuging the suspensions at 1000× *g* for 1 min. The sedimentation of the ACN aggregates results in the formation of a stable suspension of small ACNs, which can be used as a suspension for ES.

Electrospinning is carried out at a high voltage between the electrodes (usually about 20 kV), but at a low current due to the high resistance of the system; usually ES power supplies have a power of several hundred watts (150–200 μA). Thus, the conductivity of the obtained fibers is a crucial parameter to avoid the electrical breakdown and failure of the power supply.

To estimate the percolation limit of ACN suspensions for ES, the conductivity/resistance of the suspensions with different ACN concentrations in 5% PCL and 5% PVP in HFIP was measured. The volume resistance of the suspensions and films is presented in Table 3. The data obtained demonstrate the aggregation and inter-particle interaction of ACNs in a 2.5% AC–5% PCL suspension compared to a more homogeneous suspension of 2.5% AC–5% PVP. Drying of the suspension resulted in a decrease in the resistance due to solvent evaporation and the increase of inter-particle contacts, which were well detected when the 2.5% AC–5% PCL suspension was drying. This effect was less pronounced when the suspension was more dispersed (e.g., 2.5% AC–5%PVP). The elimination of ACN aggregates after centrifugation and the decrease in the ACN concentration did not reveal considerable changes in the resistance after suspension drying. In any case, suspensions with a resistance higher than 2000 kΩ are convenient for electrospinning, considering Ohm’s law. Actually, considering the distance between electrodes and the fiber thickness, the estimated electric current strength is less than 10 μA and will not overload the ES power supply or lead to electrical breakdowns.

### 2.2. Preparation and Characterization of ACN-Enriched Scaffolds

The different scaffolds were prepared using ES on a drum collector from solutions containing PCL, HSA, PVP and ACN suspensions. The electrospinning conditions and physical properties of the ACN-enriched scaffolds are summarized in Table 4. The tensile strength of the scaffolds, which varies from 2.58 to 4.65 MPa, depends on their composition and displays a typical two-phase curve with an extended plastic deformation region (Table 4). These properties provide elongation without breakage during stent installation and expansion, as previously discussed [27].

**Figure 2:**
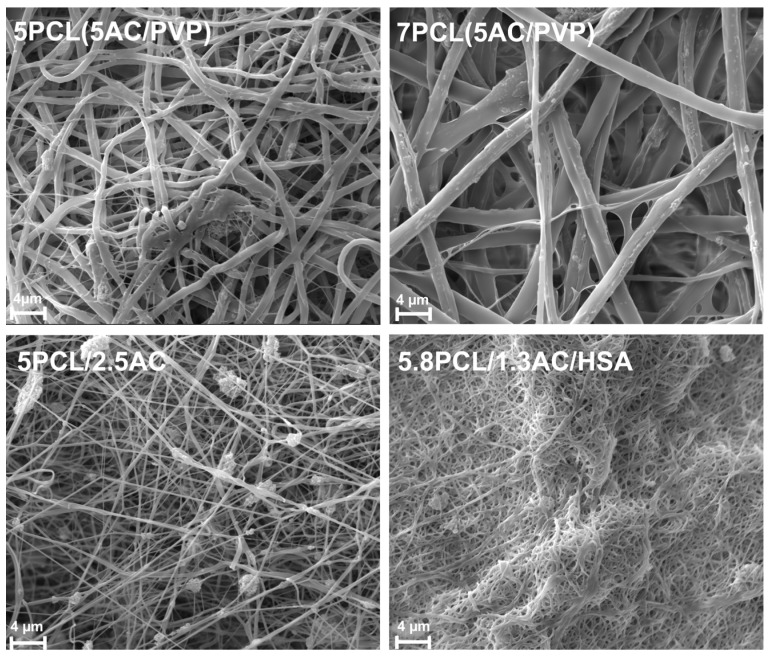
SEM images of ACN-enriched ES-produced scaffolds.

The structure of the scaffolds was studied by SEM. Depending on the ES suspensions used, the scaffolds differ in the diameter of the fibers (Figure 2). The scaffolds with homogeneous fibers (5PCL/2.5AC and 5.8PCL/1.3AC/HSA) have fibers 10 times smaller in diameter than coaxial fibers. The scaffold of 5.8PCL/1.3AC/HSA has very fine fibers. SEM image of the scaffold of 5.8PCL/1.3AC/HSA obtained at a lower magnification (×1000) shows inclusions of bulk aggregates of AC particles. The scaffold of 5.8PCL/1.3AC/HSA was difficult to obtain and manipulate since the ACNs formed large aggregates, clogged the nozzle hole during electrospinning and created stress points on the surface of the matrix. Such scaffolds are less preferable as a support for endothelial cell growth. Prior research showed that HUVEC cells cultivated on PCL-based scaffolds with fibers 2.7 µm in diameter demonstrated a maximum rate of cell adhesion and proliferation compared with fibers 0.4 µm in diameter [25,28]. PCL scaffolds consisting of fibers with a low packing density showed improved cell viability, proliferation and infiltration compared to tightly packed scaffolds [29]. Based on these data, the scaffold of 5.8PCL/1.3AC/HSA does not seem to be suitable for inner vascular stent coating. However, the introduction of an additional layer with improved biocompatibility and endothelization can overcome the disadvantages of such scaffolds.

**Figure 3:**
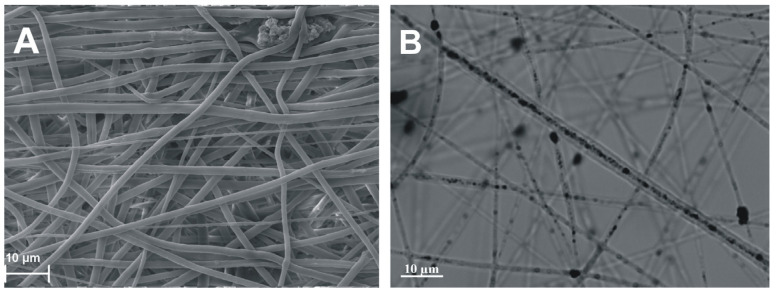
Fine structure of ACN-enriched scaffold 7PCL(5AC/PVP). (**A**) SEM image of scaffold. (**B**) Optical microscopy of scaffold on an Axiovert 200 microscope (Carl Zeiss, Germany), magnification ×2000 with immersion.

The structure of the coaxial fibers was also confirmed by optical microscopy. Figure 3B demonstrates the presence of ACNs in the inner volume of coaxial fibers.

**Figure 4:**
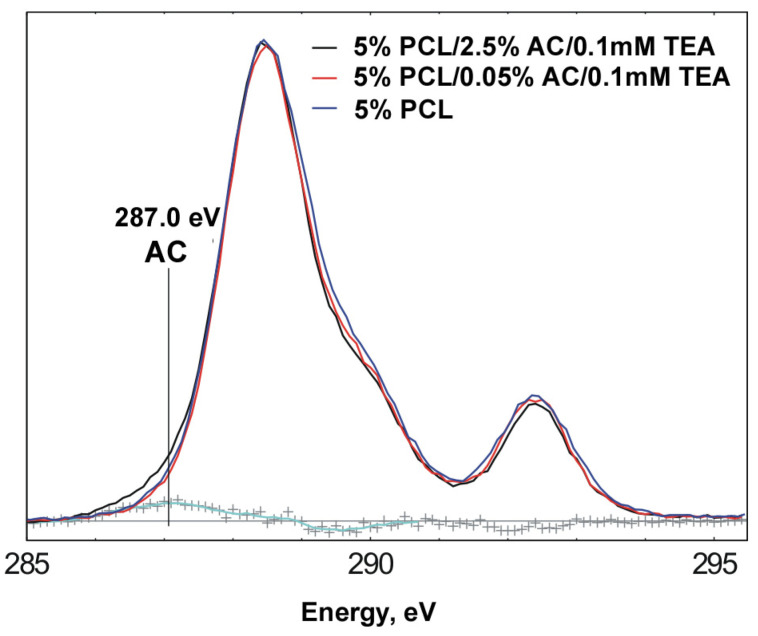
Comparison of high-resolution C1s spectra for PCL-based scaffolds: 5% PCL, 5% PCL/0.05% AC/0.1mMTEA, 5% PCL/2.5% AC/0.1mMTEA. Cyanide curve (marked as +++) corresponding to the difference spectrum between the curves for samples 5% PCL/0.05% AC/0.1mMTEA and 5% PCL/2.5% AC/0.1mMTEA.

The chemical structure of a 10 nm surface layer of scaffolds with homogeneous fibers was studied using X-ray photoelectron spectroscopy (XPS) (Figure 4, Table 5). A scaffold containing 5% PCL and 0.05% AC was also created to evaluate the effect of the ACN load on its distribution in fibers. It was found that scaffolds of 5% PCL, 5% PCL/0.05% AC and 5% PCL/2.5% AC have similar characteristics. The signal corresponding to nitrogen in the scaffold of 5PCL/2.5AC is due to nitrogen-containing groups in the ACNs [20]. As can be seen from the comparison of the spectra (Figure 4), the samples of 5% PCL and 5% PCL/0.05% AC/0.1mMTEA practically do not differ. Yet, in the spectrum of the 5% PCL/2.5% AC/0.1mMTEA sample, there is a small difference in ~287.0 eV, which can be attributed to the contribution from the ACNs. The figure also shows the difference spectrum between the curves for the samples of 5% PCL/0.05% AC/0.1mMTEA and 5% PCL/2.5% AC/0.1mMTEA (cyanide curve). The area of the difference peak is 3.1% of the area of the full peak of C1s. This value can be considered another estimate of the ACN content in the sample of 5% PCL/2.5% AC/0.1mMTEA. The comparison of the high-resolution C1s spectra for these samples revealed that the ACN content in the surface (up to 0.01 µm deep) layer of the fibers does not exceed 3.1% by weight, while the percentage of ACNs in the scaffold is 33% by weight. These data demonstrate that ACNs are distributed unequally across the diameter of the fiber; at the least, ACNs are mainly coated with a polymer layer and are not exposed on the surface of the fibers as “free” ACNs. It should be noted that covering nanoparticles with polymers is typical for fibers produced from suspensions of nanoparticles by electrospinning [30]. Thus, we can assume that in a scaffold with homogeneous ACN-enriched fibers, such as 5PCL/2.5AC AC, the nanoparticles are located in the bulk and mainly covered with a PCL layer. Covering ACNs with a polymer layer may prevent the direct interaction of the ACNs with large molecules in the blood and increase the efficacy of the ACNs’ interaction with the small molecules that readily diffuse through the pores left by the solvent evaporation in the fibers’ polymer base.

### 2.3. Study of Sirolimus Adsorption

**Figure 5:**
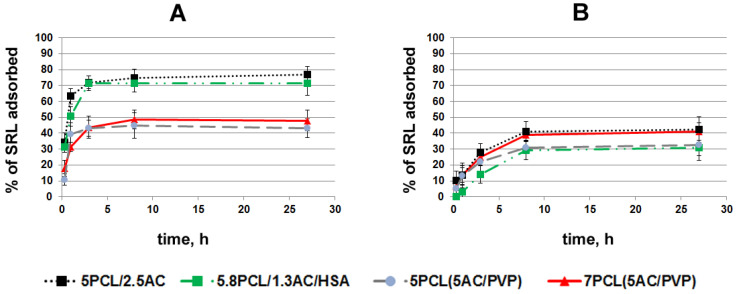
Kinetic curves of SRL adsorption onto ACN-enriched scaffolds in PBS (**A**) and in BP (**B**).

In order to select the scaffolds that most effectively bind and slowly release drugs, a study of sirolimus (SRL) adsorption onto ACN-containing scaffolds was conducted. Scaffolds were incubated with H^3^SRL in PBS or BP in order to evaluate the influence of BP proteins and low-weight molecules (lipids, etc.) on the binding of the drug with PCL-coated ACNs. Disks of 10 mm size were cut from ACN-enriched scaffolds and incubated in PBS or BP containing SRL. The ACN amounts were 0.30 ± 0.07, 0.25 ± 0.07, 1.20 ± 0.15 and 0.65 ± 0.10 mg per disc for the scaffolds of 5PCL (5AC/PVP), 7PCL (5AC/PVP), 5PCL/2.5AC and 5.8PCL/1.3AC/HAS, respectively. Scaffolds with homogeneous fibers of 5PCL/2.5AC, 5.8PCL/1.3AC/HSA absorb SRL in PBS better than 7PCL (5AC/PVP) and 5PCL (5AC/PVP) with coaxial fibers (Figure 5A). The rate of SRL adsorption in BP onto scaffolds decreases in row 5PCL/2.5AC ~ 7PCL (5AC/PVP) > 5PCL (5AC/PVP) ~ 5.8PCL/1.3AC/HSA (Figure 5B). The maximal SRL adsorption onto the scaffold of 5.8PCL1.3AC/HSA was 72% in PBS and 32% in BP, which is 45% of its capacity for SRL in PBS. For the scaffold of 7PCL (5AC/PVP), the difference in the SRL adsorption between PBS and BP was minimal (49% and 41%, respectively).

In a previous study, we presented the data on SRL adsorption onto ground AC in similar conditions [20]. The maximum SRL adsorption onto ground AC was 70–80% in PBS and 30–40% in BP. Here, we can conclude that the covering of ACNs with a PCL layer has no direct effect on the SRL interaction with ACNs because the maximum binding of SRL in PBS is very similar in terms of the maximum adsorption (up to 77%). The slight decrease in the rate of the SRL adsorption is obviously explained by the SRL diffusion through the PCL in fibers, unlike the AC suspension previously described in [20]. The work [10] describes that the adsorption of phenanthrene on composite material consisted of superfine, powdered, activated carbon (SPAC) and fibrous polystyrene (PS). It was shown that the co-spinning of SPAC and PS enhanced the adsorption capacity of the SPAC and PS when evaluated separately. It should be noted that PS itself can adsorb phenanthrene.

As we demonstrated earlier, grinding AC results in the increase of the Langmuir constant due to the functionalization of the AC. Considering the interaction of serum proteins with SRL and AC, we can conclude that BP molecules do not block so much the pores of the AC for the access of SRL, but rather reduce the binding constants of the SRL molecules with adsorption centers [20]. The packing of AC particles in PCL fibers practically does not interfere with the SRL adsorption onto ACNs packed in the fibers as compared to pure ACNs in BP. Actually, pores in electrospun-produced PCL fibers arising from solvent evaporation are easily permeable to small molecules, but not to large proteins [31], and thus, high-molecular components of BP could not interfere with the ACNs packed in the fibers.

The comparison of kinetic curves revealed that the scaffolds of 7PCL (5AC/PVP) and 5PCL/2.5AC effectively adsorb SRL in BP and may be used in biological conditions.

### 2.4. Study of SRL Release

**Figure 6:**
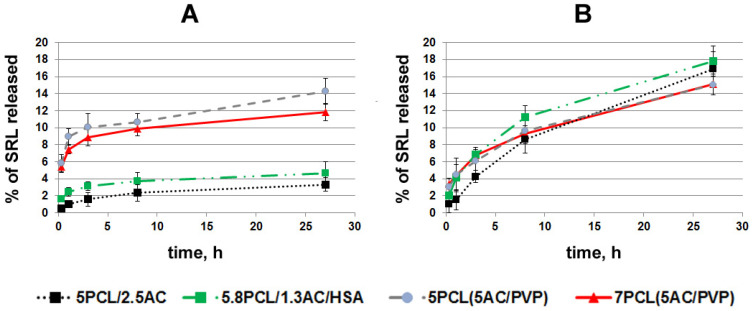
Kinetic curves of SRL release in PBS (**A**) and in BP (**B**). For adsorption of SRL, discs were incubated with SRL 10 μg/mL for 48 h in PBS or in BP. Then, supernatant was removed, and fresh PBS or BP was added. Scaffolds were incubated in PBS (**A**) or BP (**B**) for 30 min, 60 min, 3 h, 9 h and 27 h.

The targeting of SRL delivery by ACN-enriched layers, as well as drug delivery from ACN-enriched scaffolds, is tightly bound with SRL release from such scaffolds, and thus, its release from various scaffolds in PBS and BP was studied. Scaffolds were pre-incubated with SRL in PBS or BP, then rinsed with PBS and filled with 1 mL of PBS or BP, correspondingly. The initial loading of SRL was different for all scaffolds, and the data presented in Figure 6 was normalized to the amount of SRL initially bound to each scaffold.

The scaffolds of 5PCL/2.5AC and 5.8PCL/1.3AC/HSA with homogeneous fibers, which most effectively bind SRL in PBS, hold SRL most firmly: less than 5% of the bound SRL was released for 27 h (Figure 6A). SRL release from the scaffolds of 7PCL (5AC/PVP) and 5PCL (5AC/PVP) with coaxial fibers was almost 3 times faster: 12% and 14.5% for 27 h, respectively. At the same time, the scaffolds binding SRL firmly practically did not release SRL for 6 h, i.e., almost 95% of the SRL remained bound to the ACNs in the scaffolds and was no longer released in PBS.

The kinetic curves of the SRL release in BP (SRL was pre-absorbed in BP) were similar for all scaffolds tested: 5PCL/2.5AC, 5.8PCL/1.3AC/HAS, 7PCL (5AC/PVP) and 5PCL (5AC/PVP) (Figure 6B). These scaffolds released from 15% to 18% of bound SRL for 27 h, and despite the fact that the rate of SRL release decreased with time, the kinetic curve did not reach a plateau during this time. As was shown earlier, under similar conditions, ground AC released 60–70% of the bound SRL for 27 h, and full SRL release in BP occurred in 4 days [20]. Thus, the ACN-enriched scaffolds released pre-adsorbed SRL at least 4.4 times slower than free ACNs.

The strong retention of SRL on scaffolds with homogeneous fibers in PBS with a more complete release into BP can be explained by the idea that BP molecules reduce the binding constants of the SRL molecules with adsorption centers in AC [20]. This pattern is less pronounced for coaxial fibers, although the release into BP is more efficient. The slower release of SRL from ACN-enriched scaffolds compared to the ACN suspension may be related to the lack of close contacts between ACNs and SRL-binding plasma molecules, such as human serum albumin (HSA) (affinity to SRL 3.99 × 105 M^−1^, concentration 35–50 g/L [32]). Indeed, the protection of ACNs in PCL fibers from interaction with blood biopolymers hinders the redistribution of SRL between binding centers on ACNs and in BP.

The obtained data demonstrate that fiber structure is important for SRL binding/release and should be optimized for the specific problem being solved. The introduction of HSA in PCL fibers was previously shown to affect the drug release from drug-enriched fibers [27], but had no effect on the binding/release of SRL from ACN-enriched scaffolds. However, the introduction of protein into the fibers does not significantly affect the binding/release of SRL and can be used to increase the hemo- or biocompatibility of the scaffolds.

### 2.5. Cultivation of Cells on ACN-Enriched Scaffolds

**Figure 7:**
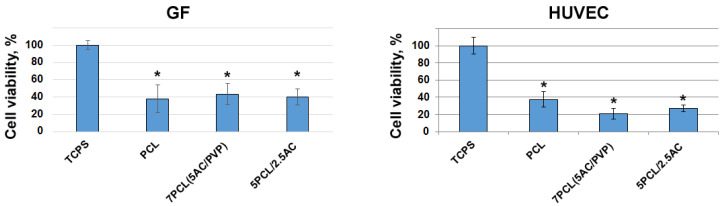
Viability of GFs and HUVECs cultured on scaffolds 48 h after seeding. Cell viability on scaffolds is represented as % to cells on TCPS. Data are shown as mean  ±  SD. * indicates statistically significant difference compared to TCPS (*p* < 0.05).

To evaluate cell adhesion and proliferation on ACN-enriched scaffolds, human gingival fibroblasts and human umbilical vein cells were cultivated in tissue culture polystyrene (TCPS) 48-well culture plates. The MTT test and AlamarBlue assay are not suitable for monitoring the viability of cells cultured on a scaffold containing ACNs, since (1) incubation of PCL scaffolds with MTT reagent resulted in the adsorption of reagent onto the scaffolds, and (2) incubation of AC-enriched scaffolds with AlamarBlue^®^ Cell Viability Reagent resulted in an increase in the absorbance at 570/600 nm, obviously due to the reduction of resazurin on AC. To measure the cell viability, we used the LDH assay after complete cell hydrolysis in 1% NP-40, which allowed us to estimate the cell viability independent from the content of the scaffolds. It was found that, compared to TCPS, less than 50% of GFs adhered to the electrospun scaffolds. The data presented in Figure 7 demonstrate that ACN-enriched scaffolds of 7PCL (5AC/PVP) and 5PCL/2.5AC have a biocompatibility for GFs similar to that in a PCL scaffold. CAN-loaded fibers did not affect the adhesion and proliferation of GFs cultured on their surface, i.e., they had a similar biocompatibility as the parental scaffolds. HUVECs are known to be more selective for support and to adhere and proliferate more poorly on ACN-enriched scaffolds. This fact must be considered when designing vascular stent coating, though it can be easily solved by introducing a thin additional hemocompatible and well-endothelized layer.

Currently, various synthetic polymers, such as poly(l-lactic acid), poly(D,L-lactide-co-glycolide) (PLGA), polytetrafluoroethylene and polyurethane, along with PCL, have been extensively studied as materials for vascular engineering. The viability of human vascular endothelial cells cultured on biodegradable polymer poly(l-lactic acid) scaffolds was 50% lower compared to cells cultivated in TCPS [33]. The adhesion and proliferation of HUVEC cells on PLGA scaffolds was 30–40% [34]. According to our data, PCL-based ACN-enriched scaffolds have demonstrated good biocompatibility in comparison with others materials.

## 3. Materials and Methods

### 3.1. Materials

The following reagents were from Sigma-Aldrich, USA: polycaprolactone (PCL, Mn 70,000–90,000 g/mol); human serum albumin (HSA); 1,1,1,3,3,3-hexafluoroisopropanol (HFIP); dimethyl sulfoxide (DMSO); polyvinylpyrrolidone (PVP, M 360,000 g/mol); trifluoroethanol (TFE); polyethylene glycol (PEG, 20,000 g/mol); polyvinyl alcohol (PVA, 89,000–90,000 g/mol); and PBS. Sirolimus (SRL) was from Fujian Kerui Pharmaceutical Co. Ltd. (Fuiqng, 150301, China). Activated carbon AX-21 was from Anderson Development Company (Adrian, MI, USA).

### 3.2. Production of 3H-SRL

Tritium-labeled sirolimus (^3^H-SRL) was synthesized by thermoactivated tritium exchange, as described earlier [35]. Following labeling, ^3^H-SRL was purified, and the radioactivity of the samples was evaluated on a Tri-Carb 2800 TR β-counter (PerkinElmer, Waltham, MA, USA) in a “ULTIMA GOLD LTT” scintillator (Perkin Elmer, Waltham, MA, USA), as reported previously [27].

### 3.3. Preparation and Characterization of the ACN

ACN particles were obtained by two-stage grinding of activated carbon AX-21 with a ball mill and then with a bead mill, as reported earlier [20].

The surface microstructure of carbon particles was studied by scanning electron microscopy (SEM). The carbon particles were fixed on a sample holder using double-sided carbon tape and analyzed using an EVO 10 scanning electron microscope (Carl 122 Zeiss AG, Oberkochen, Germany) at an accelerating voltage of 10 kV.

### 3.4. Preparation of ACN Suspensions and Estimation of a Percolation Limit

To prepare a suspension of ACNs with polycaprolactone, a 5% (*w*/*v*) solution of PCL in HFIP with 0.1 mM triethylamine (TEA) was preliminarily prepared. The ACN sample was mixed with a solution of polycaprolactone to obtain a 2.5% (*w*/*v*) ACN blend and left under stirring at room temperature on a rotary mixer at 20 rpm for 24 h, followed by treatment in an ultrasonic bath for 1 h and additionally dispersed by extrusion from a Luer lock syringe into a second similar syringe through a needle interconnecting them (needle 21G).

To prepare a suspension of ACNs with polyvinylpyrollidone, a 5% (*w*/*v*) solution of PVP in HFIP was preliminarily prepared. A weighed portion of coal was mixed with a PVP solution to obtain a 5% (*w*/*v*) ACN blend and left under stirring at room temperature on a rotary mixer at 20 rpm for 24 h, followed by treatment in an ultrasonic bath for 1 h and inter-syringe dispergation. After obtaining a homogeneous suspension, centrifugation was performed at 1000 *g* for 1 min. Gravimetry to determine the weight of sediments was performed on an Ohaus Pioneer^TM^ microbalance with an accuracy of 0.1 mg. The resulting supernatant was used for the inner layer of coaxial electrospinning.

Cast films were prepared by applying 250 µL of the ES solutions described above per 1.5 cm^2^ of glass surface. Copper electrodes 10 mm wide were used to measure the conductivity of suspensions and cast films by placing the electrodes on the surface of suspensions or films at a distance of 10 mm.

The conductivity of the solution was measured using the same electrodes in the film until it dried. An ohmmeter (V7-22A voltmeter, Radiopribor, Vladivostok, Russia) of the 2nd accuracy class was used for measurements.

### 3.5. Preparation of the Scaffolds by Electrospinning

Electrospinning solutions were prepared using PCL or PVP. They were blended with ACNs to obtain stable suspensions in HFIP at a concentration that is given as weight/volume, according to Table 4.

The list of solutions and electrospinning conditions is given in Table 1. To obtain coaxial fibers, a coaxial die with an internal needle with a diameter of 0.9/1.2 mm (internal/external) and an external one with an internal diameter of 2.5 mm was used. A stainless steel capillary with an inner diameter of 0.9 mm was used to obtain homogeneous fibers.

To obtain more uniform PCL fibers, ES solution containing 0.1mMTEA was used. The electrospun conditions are given in Table 1. The capillary-collector distance in all conditions was 20 cm. Scaffolds of a thickness of 100–120 µm were prepared using a drum collector 2 cm in diameter and 5 cm in length (30 cm^2^), rotating at 300 rpm. After fabrication, scaffolds were removed from the collector, dried under fore vacuum pressure of 10 Pa for 12 h and stored in zip-lock polyethylene containers.

### 3.6. Characterization of AC-Enriched Scaffolds

Strain–stress diagrams were obtained using a universal Zwick/Roell Z100 (Zwick Roell, Ulm, Germany) test bench, as described in ISO 7198:2016 [36]. Electrospun matrices were cut into 10 mm × 50 mm rectangular shapes and placed between holders at a distance of 2–2.5 cm. Tensile strength testing was conducted at a rate of 10 mm × min^−1^ at room temperature (21–23 °C). The thickness of the scaffolds was measured by an electronic outside micrometer (0–25 mm, 0.001, Schut Geometrical Metrology, Groningen, Netherlands 908.750), and the linear dimensions were measured using a caliper of the second accuracy class.

At least four specimens of each sample were tested.

The microstructure of the matrix surface was studied by scanning electron microscopy (SEM), as described earlier [35]. The fiber diameter and pore size were evaluated using the SEM images, according to ISO 7198:2016 [36].

To assess the stability of the fiber structure, the scaffolds were incubated in PBS or human blood plasma (BP) at room temperature for 10 days. After the incubation, the scaffolds were rinsed with H_2_O, air-dried and examined by SEM.

The porosity of the scaffolds was evaluated using scaffold volume and PCL density according to the formula: Porosity (%) = [1 − Da/Dp] × 100, where Da is the apparent density (scaffold weight/scaffold volume), and Dp is the polymer density [36].

### 3.7. Study of SRL Binding and Release

To evaluate the SRL absorption, 10 mm disks were excised from the scaffolds by die cutting and placed in the wells of a 48-well plate. The disks were covered with 400 μL of PBS or EDTA-stabilized BP containing 10 μg/mL SRL. The work was approved by the Local Ethical Committee of the Center of Personalized Medicine ICBFM SB RAS (No 8, 07.07.2020). H^3^SRL was combined with unlabeled SRL to reach a concentration of 10 μg/mL and radioactivity of 13,500 cpm/mL. The plate was sealed with a sealed adhesive film (Microseal^®^ ‘B’ PCR Plate Sealing Film, adhesive, Bio-Rad, Hercules, CA, USA) to prevent drying, followed by incubation on a Titramax 1000 shaker (Heidolph, Schwabach, Germany) at 37 °C and a platform rotation speed of 200 rpm. The scaffolds were incubated in PBS or BP for 30 min, 60 min, 3 h, 9 h and 27 h. The radioactivity of the supernatants was measured in duplicate, as mentioned in 2.2. The concentration of SRL in the solution was calculated from the specific radioactivity of the preparation.

To study the SRL release, the scaffolds with SRL adsorbed for 48 h, as described above, were washed with PBS, filled with 1 mL of PBS or BP, correspondingly, and sealed with a sealed adhesive film to prevent evaporation. It should be mentioned that rinsing the scaffolds with PBS after incubation with SRL does not noticeably decrease SRL binding. All experiments were performed in duplicate.

### 3.8. Biocompatibility of the Scaffolds

Gingival fibroblasts (GFs) obtained from human gingiva, as described in [37], were kindly gifted by Dr. Cherepanova. Human umbilical vein endothelial cells (HUVECs) were isolated and cultivated, as described previously [34,38]. Cells were cultured in IMDM (Invitrogen, Carlsbad, CA, USA) medium supplemented with 10% fetal bovine serum (FBS, Gibco, USA) and 100 units of streptavidin/penicillin in a CO_2_ incubator at 37 °C and 5% CO_2_.

To obtain a calibration curve, different numbers of cells were seeded into 48-well plates (without matrix) and cultivated for 48 h in 300 µL of IMDM-FBS. The culture medium was removed 48 h later, cells were washed twice with 100 µL PBS and then cells were lysed in 100 µL of buffer consisting of 1% Nonidet P40 (NP40, Fluka, Buchs, Switzerland) in PBS for 10 min at 25 °C. Then, the solution was collected in 1.5 mL tubes and centrifuged at 13,000 rpm for 10 min at room temperature to remove any cell debris, and the supernatant was transferred to clean tubes. To measure the LDH activity in cell lysate, 10 µL of each sample was added to 200 µL of reagent solution (0.24 mM NADH, 1.6 mM pyruvate, 0.2 M NaCl and 0.07 M Tris, pH = 7.4, Vector-Best, Novosibirsk, Russia) and incubated for 20 min at 37 °C. The reaction was stopped by adding 6 µL of 100 mM sodium oxalate (Reachem, Moscow, Russia), then the sample was transferred to a cuvette, and the absorbance at 340 nm was measured using a Genesys 10uv spectrophotometer (Thermo Electron Corporation, Waltham, MA, USA). Each sample was tested in duplicate. A calibration curve was constructed to determine the number of cells on the samples.

Disks of 10 mm diameter size were cut out from the scaffold, placed in a 48-well plate and pressed down to the bottom with polytetrafluoroethylene rings. For sterilization, plates with matrices were UV-irradiated at 280 nm for 30 min. The scaffolds were pre-incubated with 100 µL of IMDM medium for 30 min to achieve complete moistening, and the medium was removed from the wells before cell seeding. The cells were seeded 5 × 10^3^ cells per well or 10 × 10^3^ cells per well (for GFs and HUVEC cells, respectively) and cultivated for 48 h in 300 µL of IMDM-FBS. The culture medium was removed 48 h later, cells were washed twice with 100 µL PBS, and then 100 µL of lysis buffer consisting of 1% Nonidet P40 in PBS was added to each well. The cells were lysed for 10 min at 25 °C. Then, the solution was collected in 1.5 mL tubes and centrifuged at 13,000 rpm for 10 min at room temperature to remove any cell debris, and the supernatant was transferred to clean tubes. The LDH activity in cell lysates was determined as described above. Each sample was tested in duplicate. Cells from two biological donors were used to check the biocompatibility for both GFs and HUVECs.

### 3.9. Statistical Processing of Data

Microsoft Excel 2010 was used to handle and process the experimental data. Statistical analyses were performed using the Statistica 7.0 package (StatSoft Inc., Tulsa, OK, USA).

## 4. Conclusions

Application of ACN-enriched scaffolds for vectorized drug delivery, as well as for long-term drug delivery, was studied. Stable suspensions of 100 nm AC particles in polymer solutions suitable for electrospinning were prepared and used for the production of scaffolds with homogeneous and coaxial ACN-enriched fibers by ES. XPS and SEM confirmed that AC particles were located in the bulk of the fibers and mainly covered with a PCL layer. SRL binding/release depends on the fiber structure and the composition of the medium and differs from that described earlier for AC, especially when SRL is released in blood plasma. ACN-enriched scaffolds do not differ from PCL scaffolds without ACNs as a support for the cultivation of GFs, but are less convenient for the cultivation of HUVEC. ACN-enriched scaffolds meet the physico-chemical properties, drug binding/retention and biocompatibility requirements; they also show promise for long-term drug release and for vectorization of drug delivery. A further study of scaffolds consisting of a set of layers with a deposited drug for subsequent release, as well as the modification of the drug release kinetics or vectorization of drug delivery, is planned.

## Figures and Tables

**Table 1:** Properties of the suspensions (% weight/volume) prepared and assessed for electrospinning.

Composition of the Suspension		Solvent				
		Ethanol	HFIP	TFE	H_2_O	Comments
PEG	ACN concentration, %	0.01–2	0.01–2	0.01–2	0.01–2	Suspensions are not stable in all ranges of concentrations
	PEG concentration, %	2–10	2–10	2–10	2–10	
	Dispersibility *	+	+	+	+	
	suspension stability **	−	±	±	−	
PVP	ACN concentration, %	0.01–2	0.01–10	0.01–2	0.01–2	Optimal suspension contained 5% PVP, 5% ACNs (*w*/*v*) in HFIP
	PVP concentration, %	2–10	2/3.5/5/7/10	2–10	2–10	
	dispersibility	+	+	+	+	
	suspension stability	−	−/±/+/+/+	+	−	
PVA	ACN concentration, %	−	0.01–2	−	−	Suspensions are not stable in all ranges of concentrations
	PVA concentration, %	2–10	2–10	2–10	2–10	
	dispersibility	−	+	−	−	
	suspension stability	−	±	−	−	
H_2_O	ACN concentration, %	2	2	2	2	Suspensions are not stable in all ranges of concentrations
	dispersibility	−	±	±	−	
	suspension stability	−	±	±	−	
PCL	ACN concentration, %	−	0.01–5	0.01–2	−	Optimal suspension contained 5–7% PCL, 2.5% ACNs (*w*/*v*) in HFIP
	PCL concentration, %	−	5/7/10	5/7/10	−	
	dispersibility	−	+	+	−	
	suspension stability	−	+	+	−	

* (−)—low dispersibility, (+)—high dispersibility. ** (−)—unstable, (±)—semistable or (+)—stable suspensions.

**Table 2:** Sedimentation of ACNs by centrifugation in different conditions.

Treatment Suspension (% Weight/Volume)	1000 g, 0.5 min, % ACN	1000 g, 1 min, % ACN	1000 g, 5 min, % ACN	10,000 g, 5 min, % ACN	12,000 g, 30 min, % ACN	Supernatant % ACN
5% AC ^P^ *–5% PVP	46.8	17.9	15.4	7.1	5.4	7.5
5% AC ^P^–5% PVP	−	−	85.8	−	−	14.2
5% AC ^W^ **–5% PVP	30.7	27.2	30	6.2	2.9	3
5% AC ^W^–5% PVP	−	−	88.5	−	−	11.5
5% AC ^W^–5% PCL	23	22.8	43.4	8	0.3	2.5
5% AC ^W^–5% PCL	−	−	89.2	−	−	10.8

* AC ^P^—ACNs ground with the bead mill in PVP solution. ** AC ^W^—ACNs ground with the bead mill in water.

**Table 3:** Electrical resistance of AC suspensions and cast films made from them.

Suspension, Composition	Suspension Resistance, kΩ/cm	Film Resistance, kΩ/cm
2.5% AC–5% PCL	860	10
2.5% AC–5% PVP	>2000	1600
5% AC–5% PVP, 1000 g, 1 min	>2000	>2000
5% AC ^P^–5% PVP, 1000 g, 1 min	>2000	>2000

**Table 4:** Electrospinning conditions and physical properties of the scaffolds.

Composition of Scaffold		Abbreviation	Content of Particles in Scaffold mg/cm^2^	Feed Rate, mL/h Inner/Outer	Voltage, kV	Strength (MPa)	Maximum Elongation, %	Fiber Diameter (µm)	Pore Diameter (µm)	Porosity (%)
coaxial fibers	5% PCL-outer 5% AC/5% PVP-inner	5PCL(5AC/PVP)	0.40 ± 0.10	1/5	14	2.58	162%	0.99 ± 0.65	1.5 ± 0.7	42%
	7% PCL-outer 5% AC/5% PVP-inner	7PCL(5AC/PVP)	0.35 ± 0.10	1/5	14	3.48	475%	2.07 ± 0.3	7.2 ± 1.3	60%
homogeneous fibers	5% PCL/2.5% AC/0.1mMTEA *	5PCL/2.5AC	1.50 ± 0.20	0.7	24	4.65	175%	0.21 ± 0.05	2 ± 0.4	37%
	5.8% PCL/1.3% AC/0.9% HSA ***	5.8PCL/1.3AC/HSA	0.80 ± 0.20	0.7	24	ND **	ND	0.12 ± 0.02	0.3 ± 0.1	75%
no AC	5% PCL	5 PCL	no AC	1	26.5	15.04	200%	0.36 ± 0.09	2.11 ± 0.34	61%

* TEA—trimethylamine. ** ND—not determined. *** HSA—human serum albumin.

**Table 5:** Composition of elements in surface and near-surface layers of fibers according to XPS.

	O/C	P/C	N/C
5% PCL	0.33	-	-
5% PCL/0.05% AC/0.1mMTEA	0.35	-	-
5% PCL/2.5% AC/0.1mMTEA	0.35	-	0.002

O/C, P/C and N/C mean the relative content in oxygen, phosphorus and nitrogen, respectively. (-)—undetectable level.
